# A Response Surface Methodological Approach for Large-Scale Production of Antibacterials from *Lactiplantibacillus plantarum* with Potential Utility against Foodborne and Orthopedic Infections

**DOI:** 10.3390/antibiotics13050437

**Published:** 2024-05-13

**Authors:** Paulpandian Prema, Daoud Ali, Van-Huy Nguyen, Bhathini Vaikuntavasan Pradeep, Veeramani Veeramanikandan, Maria Daglia, Carla Renata Arciola, Paulraj Balaji

**Affiliations:** 1 Department of Zoology, VHN Senthikumar Nadar College, Virudhunagar 626001, TN, India; prema.drprema@gmail.com; 2 Department of Zoology, College of Science, King Saud University, P.O. Box 2455, Riyadh 11451, Saudi Arabia; aalidaoud@ksu.edu.sa; 3 Centre for Herbal Pharmacology and Environmental Sustainability, Chettinad Hospital and Research Institute, Chettinad Academy of Research and Education, Kelambakkam 603103, TN, India; vhnguyen.che@gmail.com; 4 Centre for Microbial Technology, Department of Microbiology, Karpagam Academy of Higher Education, Coimbatore 641021, TN, India; pradeep.bv@kahedu.edu.in (B.V.P.); vra.manikandan@gmail.com (V.V.); 5 Department of Pharmacy, University of Naples Federico II, Via D. Montesano 49, 80131 Naples, Italy; maria.daglia@unina.it; 6 International Research Center for Food Nutrition and Safety, Jiangsu University, Zhenjiang 212013, China; 7 Laboratory of Immunorheumatology and Tissue Regeneration, Laboratory of Pathology of Implant Infections, IRCCS Istituto Ortopedico Rizzoli, Via di Barbiano 1/10, 40136 Bologna, Italy; 8 Department of Medical and Surgical Sciences (DIMEC), University of Bologna, Via San Giacomo 14, 40126 Bologna, Italy; 9 PG and Research Centre in Biotechnology, MGR College, Hosur 635130, TN, India

**Keywords:** Box–Behnken design, bacteriocins, *Lactiplantibacillus plantarum*, antibacterial activity, orthopedic implant infections, foodborne infections

## Abstract

A variety of bacteria, including beneficial probiotic lactobacilli, produce antibacterials to kill competing bacteria. Lactobacilli secrete antimicrobial peptides (AMPs) called bacteriocins and organic acids. In the food industry, bacteriocins, but even whole cell-free supernatants, are becoming more and more important as bio-preservatives, while, in orthopedics, bacteriocins are introducing new perspectives in biomaterials technologies for anti-infective surfaces. Studies are focusing on *Lactiplantibacillus plantarum* (previously known as *Lactobacillus plantarum*). *L. plantarum* exhibits great phenotypic versatility, which enhances the chances for its industrial exploitation. Importantly, more than other lactobacilli, it relies on AMPs for its antibacterial activity. In this study, Response Surface Methodology (RSM) through a Box–Behnken experimental design was used to estimate the optimal conditions for the production of antibacterials by *L. plantarum*. A temperature of 35 °C, pH 6.5, and an incubation time of 48 h provided the highest concentration of antibacterials. The initial pH was the main factor influencing the production of antibacterials, at 95% confidence level. Thanks to RSM, the titer of antibacterials increased more than 10-fold, this result being markedly higher than those obtained in the very few studies that have so far used similar statistical methodologies. The Box–Behnken design turned out to be a valid model to satisfactorily plan a large-scale production of antibacterials from *L. plantarum.*

## 1. Introduction

Lactobacilli act as “health-friendly bacteria” with beneficial antagonistic effects against pathogenic bacteria through a variety of mechanisms, including the secretion of the following antibacterial molecules: organic acids, such as lactic acid, acetic acid, and formic acid; biosurfactants; hydrogen peroxide; and last, but not least, small ribosomal antimicrobial peptides, the so-called bacteriocins [1,2]. Antimicrobial peptides (AMPs) are innate immunity molecules first identified in humans within neutrophil granules [3] and, more recently, also recognized as fratricide molecules generated by bacteria against other bacteria. Neutrophils, the first line of innate immune defense against pathogens, use the AMPs of their granules to defend human tissues from bacterial infections [4]. Bacteria living in polymicrobial communities produce AMPs to attack other bacteria that compete with them for essential resources or otherwise threaten their survival or well-being [5]. *Lactobacillus* is the largest genus within the group of lactic acid bacteria. Different species belonging to the *Lactobacillus* genus (recently reclassified as *Lactiplantibacillus* [6]) reside on human mucous membranes where they play a role as probiotics [7]. Importantly, both cell-free supernatants and isolated bacteriocins by *Lactiplantibacillus* demonstrated antibacterial properties against the harmful infectious bacterial agents that can contaminate foods, such as *Staphylococcus aureus* [8], *Escherichia coli* [8,9], *Pseudomonas aeruginosa* [10], *Bacillus cereus* [11], *Listeria monocytogenes* [12]. 

*L. plantarum* is a versatile Gram-positive lactic acid bacterium, originally found in saliva, belonging to the large family of *Lactobacillacae. L. plantarum* strains isolated from various environmental niches exhibit phenotypic and genotypic diversities [13]. Recently, Carpi et al. conducted a comprehensive pan-genome analysis of *L. plantarum* demonstrating that this bacterium is endowed with one of the largest genomes known among the lactic acid bacteria [14].

For its antimicrobial properties, *L. plantarum* seems to rely, more than other lactobacilli, on antimicrobial peptides, which makes it even more interesting, given the great potential of these molecules. In this connection, Arrioja-Bretón et al. demonstrated that the antimicrobial activity of CFS from various bacterial species was lost by adjusting the pH to 6.5, with only CFS from *L. plantarum* retaining its antimicrobial activity, which was instead lost by treating with proteinase K [15].

Most of the bacteriocins produced by *L. plantarum* generally belong to Class II bacteriocins and are collectively referred to as plantaricins. They are non-lantibiotic, small (<10 kDa) two-peptide molecules, hydrophobic, cationic, unmodified, and stable to heat [16]. Plantaricins may be either chromosomally or plasmid encoded and are usually organized in operon clusters [17].

Bacterial species such as *S. aureus*, *E. coli*, *P. aeruginosa*, and *Bacillus cereus*, which have a high prevalence as food contaminants, are increasingly considered “One Health” threats [18]. Remarkably, many of these bacterial species coincide with the species that cause post-surgical orthopedic infections. Indeed, the Gram-positive bacterium *S. aureus* and the two Gram-negative bacteria *E. coli* and *P. aeruginosa* are, one, the main etiological agent of orthopedic implant infections and, two, among the bacteria that most commonly infect the periprosthetic bone tissues [19]. *B. cereus* is a significant pathogen in postoperative and post-traumatic wounds in orthopedic wards [20,21] and in infections related to orthopedic implants [22]. Noticeably, most AMPs act by piercing the bacterial envelopes with consequent depolarization and destabilization and, finally, disruption of the bacterial plasma membrane, causing bacterial cell death [23]. Therefore, unlike antibiotics, they do not evoke bacterial resistance. Most LAB-bacteriocins (bacteriocins produced by lactic acid bacteria) act by disturbing the cytoplasmic membrane through forming pores or by cell wall degradation [24]. Nevertheless, some of these peptides still have unknown modes of action, especially those that are active against Gram-negative bacteria [24]. *L. plantarum*-derived plantaricins have been shown to be effective against Gram-positive bacteria, such as *S. aureus*. Certain plantaricins are effective against Gram-negative bacteria, such as *E. coli* [24]. 

It is not clear how the probiotic properties of *L. plantarum* are mediated by quorum sensing [25]. A recent study suggests that the enhancement of the AI-2/*LuxS* quorum sensing of *L. plantarum* enables the regulation of the interspecific bacterial communication useful to reduce and then eradicate biofilms of putrefying bacteria [26]. 

Another interesting study suggests that bacteriocin synthesis by *L. plantarum* co-cultured with other bacterial species might be controlled by the *LuxS*-mediated quorum sensing system of *L. plantarum* [27].

Bacteriocins appear to promise new applications in clinical fields where infections are still an unsolved problem. Infections associated with orthopedic implants are a frequent cause of implant failure. Zhou et al. focused on bacteriocins as potential agents for the prevention of post-surgical infections of orthopedic implants. They demonstrated the antibacterial effects of bacteriocins from *Lactobacillus rhamnosus* in a rabbit model of knee prosthesis infection by *S. aureus* [28]. Biomaterials with infection-resistant surfaces represent an important strategy to prevent or combat orthopedic implant infections. Lallukka et al. functionalized the surface of a titanium alloy, a common material to a variety of orthopedic implants, using the bacteriocin nisin and documented the antibacterial activity of the nisin-modified surface against a methicillin-resistant *S. aureus* strain [29].

Very recently, studies have focused on the promising probiotic species *L. plantarum* and on cell-free supernatants and bacteriocins produced by different strains of *L. plantarum.* Wang et al. demonstrated the utility of the *L. plantarum* 90 (LCFS) as an antibacterial agent to improve the shelf life of ground meat gel [30]. Li et al. showed a marked decrease in *S. aureus* counts in sausages inoculated with *L. plantarum* SL47 and its bacteriocin SL47 [31]. Huang et al. demonstrated the antimicrobial potential of *L. plantarum* strains TE0907 and TE1809 from the Asian toad Bufo gargarizans [32]. Xu et al. demonstrated the anti-infective effect of a combination of tea polyphenols and *L. plantarum* ST8SH bacteriocin in a rabbit model of infection following femoral fracture with internal fixation [33].

Peculiar metabolic activities of *L. plantarum* are important for industrial applications. The metabolic characteristics that are intrinsic of *L. plantarum* are the reason for their versatility and success in industrial applications. Some strains of *L. plantarum* are known to produce various natural antimicrobial compounds, γ-aminobutyric acid (GABA), and exopolysaccharides (EPSs) and to exhibit antioxidant and β-glucosidase activities. Moreover, *L. plantarum* is “Generally Recognized As Safe” (GRAS) by the US Food and Drug Administration (USFDA) with “Qualified Presumption of Safety” (QPS) from the European Food Safety Authority (EFSA) [34].

*L. plantarum* is also recognized as an able enhancer of food safety during the fermentation process [35], and it has proven to be a valuable species for the development of probiotics [36,37]. 

Finally, as mentioned above, *L. plantarum* is endowed with more genes than other lactobacilli, thus highlighting its multifaceted versatility and the variety of their phenotypes, with consequent great potentiality of industrial applications [16,17]. 

In consideration of all the above, it would be very useful to have valid methods to optimize the production of *L. plantarum* antimicrobials.

Response Surface Methodology (RSM) is a combination of statistical and mathematical techniques to predict the effect of several independent variables by building a model to recognize the optimal values of the variables. RSM lends itself to being exploited for the modeling and optimization of a wide variety of microbial products [38,39]. Box–Behnken designs (BBDs) are experimental designs for RSM. This methodology has also been regarded as more economical than a full multifactor experiment when the predictor variables are greater than two [40,41,42,43,44,45,46,47,48]. One of the most important aspects in the study of bacteriocin is its production. Indeed, the production of bacteriocin is, in general, very low and the costs of producing it in large quantities for industrial use are high. Moreover, the production of antibacterials is influenced by several species-specific (or even strain-specific) variables. The aim of the present study was to optimize the production of antibacterials by a strain of *L. plantarum* using an experimental BBD. The relationship between the operational variables (incubation temperature, pH, incubation period) was described in a statistically significant quadratic model. The results revealed that the model could be useful for large-scale production of antibacterials, with potential application prospects.

## 2. Results

### 2.1. Optimization Parameters for Antibacterial Production

To optimize the three operational variables (temperature, pH, and incubation time) to maximize bacteriocin production, a BBD for RSM was used. Table 1 shows the coded values as well as the real experimental concentrations of selected independent variables.

The design matrix included 17 runs and experimental responses, with bacteriocin production ranging from 350 to 3650 AU/mL. With optimum operational parameters of 35 °C temperature, 6.5 pH, and 48 h of incubation, the maximum bacteriocin score (3650 AU/mL) was observed in run 15 (Table 2), indicating that the experimental and predicted bacteriocin values were close to each other. Multiple regression analysis was used to examine the bacteriocin responses, as well as the correlation between the projected responses and factors as stated by a quadratic polynomial expression:

*Bacteriocin activity* = +3530.00 + 62.50*A* − 128.12*B* + 3.12*C* + 250.00*AB* − 75.00*AC* + 156.25*BC* − 1311.88*A*^2^ − 1418.13*B*^2^ − 1543.13*C*^2^ (1)

The findings from the one-way ANOVA analysis indicated that the three independent variables had a significant impact on the bacteriocin activity (Table 3). The significance of the experimental data of the various models was determined using ANOVA. The relevant *p*-values are listed in the aforementioned table. Three linear coefficients (A, B, and C), three quadratic coefficients (A^2^, B^2^, and C^2^), and two interactive coefficients (AB) were statistically significant, reflecting the interactive effects between the tested variables of each model term. The obtained F-value (120.25) suggests that this was highly significant (*p* < 0.001). There is only a 0.01% chance of that the predicted model F value would occur due to noise. The “Lack of Fit F-value” of 4.54 indicates there is a 8.90% chance that could occur due to noise. Overall, the model is highly fitted, and the optimization parameters used in the experiment lead to a predicted good yield and do not obtain poor misleading results. The probability value is significant for each coefficient, and the intensity of the interactive effects between the variables is considered significant (*p* < 0.05). The stronger the connection between the observed and expected values, the greater the significance value. Furthermore, the mathematical model has been adjusted effectively, as evidenced by a good coefficient of determination (R^2^ = 0.9936). In comparison to the fitted model, the ANOVA of the quadratic polynomial model is given greater significance due to a higher F value (120.25; *p* < 0.0001). The fitness R^2^ value 0.9936 of the model prediction reflects variations in the parameters (99.4%), and only 0.6% of the total variance cannot be explained using this model. Based on the obtained data, the bacteriocin production parameters including temperature and initial pH, as well as their interactions, have a statistically significant effect on bacteriocin production (*p* < 0.05). The individual variables (temperature, initial pH, and incubation time) and interactions such as A^2^, B^2^, and C^2^ show a greater significance on bacteriocin activity (*p* < 0.0001). Moreover, independent variables such as A, B, and C result in no increase in bacteriocin production (*p* > 0.05).

**Figure 1:**
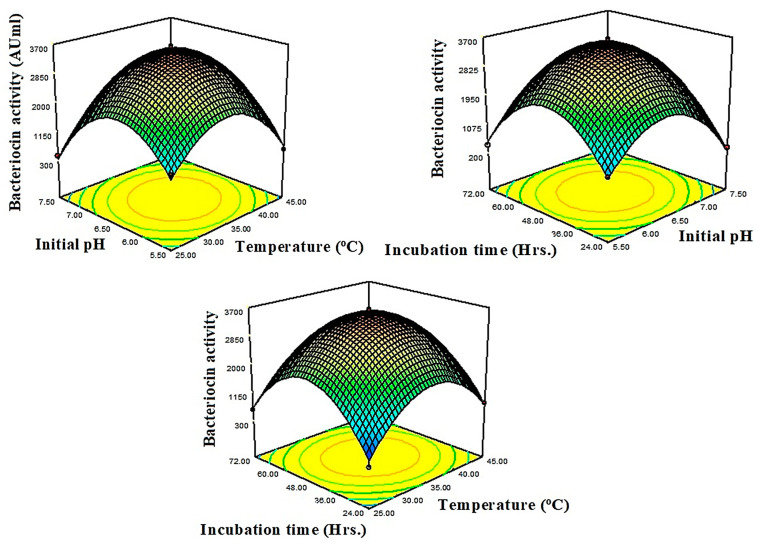
Response surface plots showing interactive effects between independent variables (temperature, pH and incubation time) for improved bacteriocin production by *Lactobacillus plantarum*.

**Figure 2:**
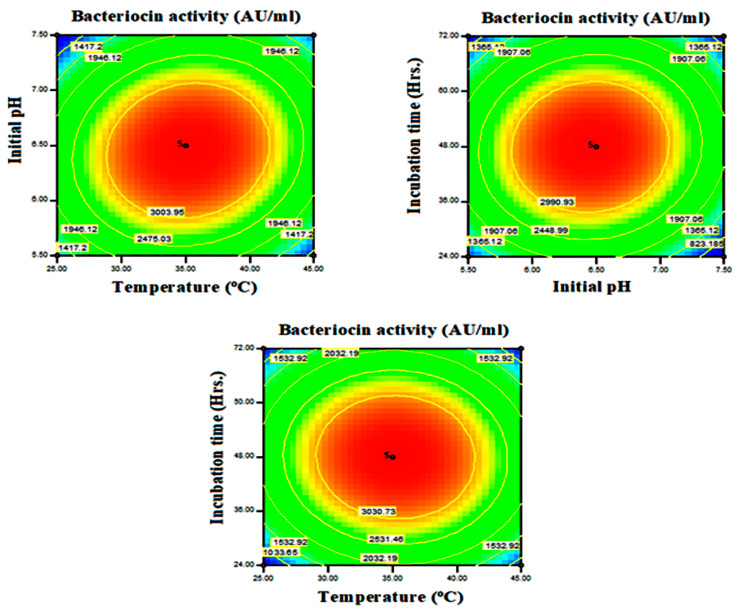
Contour plots showing interactive effects between study parameters for enriched bacteriocin production by *Lactobacillus plantarum*.

**Figure 3:**
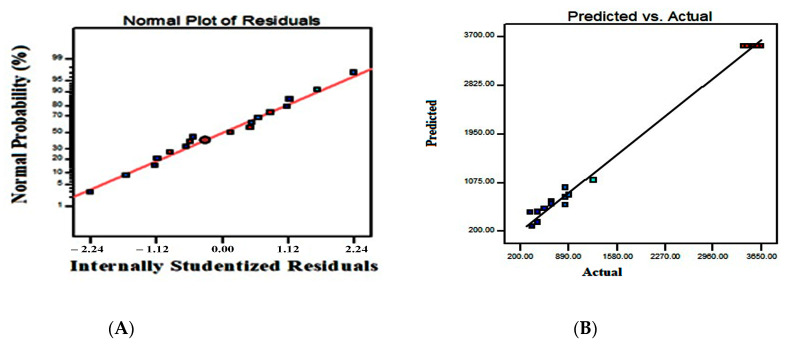
Normal probability plot of the residuals of a regression model for increased bacteriocin production by *Lactobacillus plantarum:* (**A**) Correlation between actual and predicted values of the quadratic polynomial model; (**B**) Antibacterial activity of crude bacteriocin.

The non-fitted F-value for the present model was 4.54. This is not a significant variation when compared with pure error. Consequently, the fitness of the quadratic model was confirmed. The adjusted determination coefficient (Adj R^2^) corrects the determination coefficient (R^2^). The adj R^2^ value may be substantially smaller than the R^2^ value, if there are numerous terms in the model, and the sample size is not very large. The difference between R^2^ (0.9936) and adjusted R^2^ (0.9853) was less than 0.2, with the strong agreement of these two values confirming the model’s strength. Contour and 3D surface plots were used to explain the relative consequence of any two parameters, the values in other variables being set to their central point values. Figure 1 shows the substantial effects exerted by study parameters such as temperature and starting pH on bacteriocin synthesis at a constant incubation time of 48 h, with the elliptical contour plot being obtained from the present findings. The initial pH of the production medium leads to an increasing trend in bacteriocin output, according to the present findings. As a result, the AB interaction (temperature vs. starting pH) provides a beneficial impact on bacteriocin activity against *B. cereus*. At a fixed initial pH of 6, the effect of synthesis process, temperature, and incubation time on bacteriocin production results in a circular contour shape, revealing a non-significant mutual effect of temperature and incubation time on bacteriocin activity, in addition to both factors’ linear effects. A positive reaction can also be provoked by changing the value of a constant variable (pH) or expanding the ranges of interacting variables (temperature vs. incubation time) (Figure 2). At 35 °C, an incubation duration of 48 h and an initial pH of 6.5 resulted in a significant bacteriocin production. Increases or decreases in the levels of two variables had a negative impact on yield. The residual graph, which provides useful information, can also be used to interpret the model fitness (Figure 3). The disparity between actual and predicted values is referred to as residuals. The comparison between normal probability (%) vs. studentized residuals revealed that meticulous values provide adequate model estimates. Furthermore, the residual plot was linear, satisfying the normality condition, which also confirms the accuracy of the experimental BBD.

### 2.2. Validation of the Model

To estimate the dispersion of empirical error terms, a homogeneity percentile plot was employed. The parameters measured followed a near-perfect linear distribution, indicating the model’s relevance and correctness. The normality assumption of empirical residues was shown by a horizontal line in this graph, confirming the model’s validity (Figure 3). Measurements were taken in five independent repetitions, with the appropriate optimum settings for evaluating the results and establishing the accuracy of the predictions made: With 48 h of incubation under steady circumstances, at a pH of the medium of 6.5, and a temperature of 350 °C. The experimental results (3650 AU/mL) agreed with the anticipated values (3530 AU/mL), demonstrating the appropriateness of the model.

**Figure 4:**
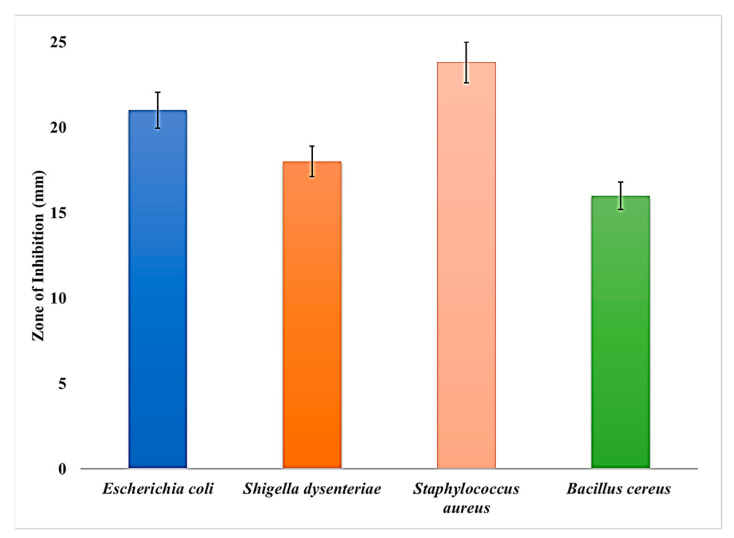
Inhibitory zone (mm) of bacteriocin produced by *Lactobacillus plantarum* against four pathogens. Each value is the mean ± SEM of five individual replicates. The differences differ significantly from each other (one-way ANOVA test; *p* < 0.001 and subsequent post hoc DMRT).

The antibacterial effects of the crude bacteriocin produced were tested against four different bacterial species: *E. coli*, *Shigella dysenteriae*, *S. aureus,* and *B. cereus*, and results are represented in Figure 4. The maximum inhibition zone of 23.80 ± 1.33 (mean ± S.D.) was obtained against *S. aureus*, followed by *E. coli* (21.00 ± 0.63). The inhibitory effect of bacteriocin was demonstrated with the zone of inhibition obtained in the experiment. The one-way ANOVA test revealed that inhibitory zones demonstrated against the bacterial pathogens are statistically significant based on F value (F = 49.76; *p* < 0.001). A post hoc analysis for the bacteriocin inhibitory effect against bacterial pathogens was accomplished using a DMR test, in addition to the ANOVA, and this showed a greater difference between the bacterial strains of the different species (Figure 4).

## 3. Discussion

The influence of several operational parameters, namely, temperature, initial pH, and incubation time, on bacteriocin production from a strain of *Lactobacillus plantarum* were assessed using the RSM approach through a BBD. Initially, the range of BBD optimization for the different parameters was determined by evaluating a single-factor effect on the overall yield. The total yield increased significantly when the process temperature, initial pH of the production medium, and incubation time were increased to 35 °C, pH 6.5, and 48 h, respectively, according to the results of the single-factor effect, but no significant increase in yield was detected when temperature, pH, and incubation time were further increased or were decreased. The increased bacteriocin activity titer of 3650 AU/mL was recorded at 35 °C, pH 6.5, and incubation time of 48 h. The data obtained from the equation indicate that the incubation temperature and pH of the model exert a stronger influence than other operational variables, at a 95% confidence level. 

Our previous research indicates that bacteriocin production begins approximately 12 h after incubation, aligning with the exponential growth phase of *L. plantarum*. Production persists for up to 72 h, peaking at 48 h. After reaching this peak, as time progresses, the production of bacteriocin decreases perceptibly. The objective of the previously published results is to offer an in-depth view of the temporal dynamics of bacteriocin production in relation to the growth phases of *L. plantarum* [49]. Similar patterns of growth and bacteriocin production were supported by research conducted by Georgieva et al. [50], Smetankova et al. [51], and by Callewaert and De Vuyst [52]. Georgieva et al. [50] observed similar growth parameters for *L. plantarum* in traditional white cheese, while Smetankova et al. [51] studied the influence of aerobic and anaerobic conditions on the growth and metabolism of selected strains of *L. plantarum*. Furthermore, Callewaert and De Vuyst [52] focused on improving and stabilizing bacteriocin production with *Lactobacillus amylovorus* DCE 471 through fed-batch fermentation. Salman et al. [39] reported on bacteriocin production by *Lactobacillus acidophilus* MS1 under BBD-optimized process conditions. The results obtained were similar to those of the present study, with the maximum amount of bacteriocin (2600 AU/mL) observed at 300 °C, pH 6, and incubation time of 18 h. Also in the above-mentioned study, the pH of the regression model production medium was found to significantly influence bacteriocin production. The above studies collectively contribute to the knowledge of the kinetics of bacteriocin production and highlight the importance of considering various factors that may influence growth dynamics and antimicrobial peptide production in probiotic bacteria.

In this specific investigation, pH variations in the culture medium were not monitored. However, our previous research [49] addressed the impact of pH on bacteriocin production. It was observed that the *L. plantarum* strain showed its maximum optical density at the wavelength of 600 nm when the pH of the environment was maintained at 6.5. This indicates that bacterial cells reach their maximum density or concentration under these specific pH conditions. Furthermore, at this optimal pH of 6.5, the final pH of the medium after bacterial growth reached the value of 3.9. This change in pH from initial to final pH reflects the metabolic activities of the bacteria during growth, including the production of organic acids and other metabolites. 

Interestingly, concomitant with the higher optical density, the same strain showed significantly increased bacteriocin activity when grown at pH 6.5. Bacteriocins are antimicrobial peptides produced by bacteria to inhibit the growth of closely related or competing bacterial species. The high bacteriocin activity observed at pH 6.5 suggests that this pH condition favors the production of these antimicrobial peptides by *L. plantarum*.

Furthermore, statistical analysis of pH changes revealed a significant difference, as indicated by a calculated F value of 53.020 with a corresponding probability value (*p* < 0.05). This statistical analysis highlights the importance of pH in influencing bacteriocin production by *L. plantarum*, as well as the reliability and significance of the differences observed in pH conditions. These findings highlight the complex relationship between pH levels in the culture medium and the growth and bacteriocin production capabilities of *L. plantarum*, providing valuable information on environmental factors that may influence the bacteriocin production of probiotic bacteria.

Salman et al. [53] reported on the bacteriocin production by *Lactobacillus acidophilus* MS1 under the optimized process conditions they recognized through a BBD. The findings they obtained were like those of the present study, with their greatest quantity of bacteriocin (2600 AU/mL) being observed at 30 °C, pH 6, and incubation time of 18 h. Even in the aforementioned study, the production medium pH of the regression model was found to significantly influence the bacteriocin production. These findings corroborate our experimental results. 

Each operational variable (A, B, and C) was assessed for its impact on the bacteriocin production using the BBD analysis. When B and C or A and C interacted, the bacteriocin activity output turned out reduced. But all independent variables squared demonstrated a positive effect on the bacteriocin production, as well as the A and B interaction, which also exerted a beneficial effect on the bacteriocin production.

There was a sound conformity between the projected R^2^ and the adjusted R^2^ and, therefore, a close connection between actual and predicted values. The difference between R^2^ (0.9936) and adjusted R^2^ (0.9853) was less than 0.2, with the strong agreement of these two values confirming the model strength. Moreover, these results agreed with those of previous studies [49,54].

A sufficiently accurate value is required to compute the quotient representing the circumstantial noise, which would be expected to be greater than 4. The value obtained in the model of the present study was 10.83, thus demonstrating that the regression indicated a positive signal and a good fit.

The experimental values lie on the 10°, indicating that the predicted values are in close agreement with the experimental ones. Similarly, previous studies [55] report that a statistical model of linear programming techniques with a ratio of 45.389 gave a positive response. The empirically measured values lie on 45°, demonstrating that the RSM model figures are well comparable, according to the goodness-of-fit values of the RSM design. The bacteriocin yield was optimized in this study using a BBD biostatistical tool. Bacteriocin production under optimized conditions (3650 AU/mL) was much higher (10.43 times) than that under non-optimized conditions (350 AU/mL). Thirumurugan et al. [56], using a statistical design to maximize the bacteriocin production by *L. plantarum* ATM11 through optimization of the medium components, achieved a 5.75-fold yield. In the study by Zhou et al. [57] the optimization of the components of the medium for the synthesis of nisin led to four times lower values in the amount of nisin compared to the bacteriocin values obtained in the present investigation. 

In the present study, we also studied the in vitro inhibitory activity of the *L. plantarum* strain on the in vitro growth of four different pathogenic bacterial species (4/4 implicated in foodborne infections and 3/4 in orthopedic implant infections), demonstrating the effective efficacy of the *L. plantarum* bacteriocin on the tested pathogens with a stronger effect against *S. aureus* and *E. coli*, two species that act as major pathogens in both foodborne and orthopedic implant infections. 

Previous studies reported on inhibitory substances produced by lactobacilli that are able to target the cell membrane of bacterial pathogens and form passage channels in their membrane, causing self-digestion and necrobiosis [58,59]. Quantitative investigation of foodborne bacterial pathogens after treatment with *L. plantarum* cell-free culture supernatant found that it served as an effective antimicrobial [30]. In an investigation, the cell-free culture supernatant of *L. plantarum* strain LA21 was effective against pathogens such as *Bacillus pumilus*, *Bacillus amyloliquefaciens*, *S. aureus*, and *L. monocytogenes* [60]. A previous study [61] reported that the treatment with a culture supernatant from *Lactobacillus brevis* reduced the vitality of *E. coli* and *Salmonella typhimurium* by about 29 and 30 percent, respectively. Other studies reported that *Lactobacillus* strains can exert good antibacterial effectiveness against *C. difficile*, *E. coli*, *Shigella* spp., *S. mutans*, *P. aeruginosa*, and *S. aureus* [62,63,64]. The activation of the PA-1 and LPL-1 genes responsible for the synthesis of a new class of bacteriocins (plantaricins) was found to be associated with the inhibition of *L. monocytogenes* [12]. Cell-free supernatants of *Lactobacillus acidophilus* isolated from yogurt had strong antimicrobial activity against *P. aeruginosa* and *Klebsiella pneumoniae* [10]. Cell-free supernatants from *Lactobacillus* species collected from healthy newborns revealed high antibacterial activity against multidrug-resistant *E. coli* [9].

Some further interesting examples follow with focus on the *Staphylococcus* genus, and, more in particular, on the *S. aureus* species. Park et al. found that *Ligilactobacillus animalis* SWLA-1 and its supernatant significantly inhibited multidrug-resistant staphylococci both in vitro and in a rat model of acute osteomyelitis, which is a severe complication of orthopedic surgery [65]. Zhu et al. purified and characterized a novel bacteriocin (plantaricin ZJ008) from *L. plantarum* ZJ008, which was active against *Staphylococcus* spp [66]. A bacteriocin produced by *Lactobacillus coryniformis* was shown to be effective against *S. aureus* and *E. coli* [8]. Peng et al. characterized a broad-spectrum novel bacteriocin produced by *L. plantarum* SHY 21–2 from yak yogurt [67]. Zhu et al. purified a bacteriocin from *L. plantarum* ZJ217, which proved effective against methicillin-resistant *S. aureus* [68]. Xu et al. demonstrated the anti-infective effect of the interesting combination of tea polyphenols and *L. plantarum* ST8SH bacteriocin in a rabbit model of staphylococcal infection following femoral fracture with internal fixation [33]. 

The action that bacteriocins from lactobacilli, especially from *L. plantarum*, express against *S. aureus* deserves special attention. And indeed, *S. aureus* is a very insidious opportunistic pathogen, capable of causing serious infections in immunocompromised patients and in prosthesis wearers (*S. aureus* is the main etiological agent of orthopedic implant infections), as well as acting as a foodborne pathogen. Moreover, it is becoming increasingly resistant to antibiotics.

Probiotic strains and bacteriocins are already present on the market. However, the search for new strategies and methods to recognize and select additional precious lactobacillary microflora and to enhance the effect of the most appropriate bacteriocins for different uses is a fruitful path to pursue [69]. This is especially true when considering that freely produced crude bacteriocin can express modest antimicrobial activity, as demonstrated against *E. coli* in the study by Pato et al. [70].

It is worth underlining that, although bacteriocins are generally considered to be non-toxic for eukaryotic cells, various assays must be performed to establish the safety of bacteriocins before their use in food and medicine applications.

## 4. Materials and Methods

### 4.1. Bacterial Strain and Growth Condition

Chemicals and culture media were procured from Hi-Media, Mumbai, India. Sterile type I water was utilized to prepare solutions and culture media. The bacterial strain of *Lactobacillus plantarum* used in this experiment was previously isolated and identified according to biochemical studies, as well as by fermentation of carbohydrate profile using an API 50 CHL system, Bio Merieux, Craponne, France [46]. The identity of *Lactobacillus plantarum* was confirmed using BLASTn, against conventional categories from the NCBI data source, and the 16 s rRNA gene sequence was found in the Genbank Database with this accession number (Accession no. MK533455). De Man Rogosa and Sharpe agar was used to maintain the *L. plantarum* strain. The colonies of *L. plantarum* strain were picked from MRS agar, and individual pure culture was obtained by repeated streaking on a MRS agar plate. The purified strain was stored at 4 °C for further studies. An individual colony was inoculated into 20 mL of MRS broth at 35 °C for 48 hrs for study of bacteriocin production. The incubated cells were kept in centrifugation at 7000 rpm for 10 min, with a density of O.D. 0.72 at 600 nm. The culture supernatant was filtered through 0.22 µm membrane filters (Millex-GV filter, Millipore, Burlington, MA, USA) and used for further assays.

### 4.2. Detection of Bacteriocin

The bacteriocin produced was detected using an agar well diffusion method [47]. Every supernatant had been tested against a test organism to determine its antibiotic effectiveness (*Bacillus cereus*, MTCC 619). Bacteriocin activity was evaluated in AU/mL (Arbitrary units per milliliter), with each AU representing one unit of antibiotic action. The inhibitory reaction zone area per unit volume (mm^2^/mL) was used to compute one AU. Five separate trials were conducted, with a standard error mean of 5%. Antibiotic activity was measured through the area of the inhibitory zone (mm^2^), applying the calculation below [48].

Inhibitory activity (mm^2^/mL) = *Lz − Ls/V*

L*z* = clear zone area (mm^2^); L*s* = well area (mm^2^); *V* = volume of sample (mL).

### 4.3. Antibacterial Activity of Bacteriocin

The modified agar well diffusion method [71] was used to detect the antimicrobial activities of culture supernatants from *L. plantarum.* Fresh overnight pathogenic bacterial cultures were used to form a lawn over the surface of Mueller Hinton agar plates. Then, crude cell-free supernatants from isolated lactobacilli were injected in four different concentrations, 12.5, 25, 37.5, and 50 µL, into each 6 mm diameter well, separately. A clear zone of inhibition was measured after overnight incubation period at 37 °C.

### 4.4. Experimental Design

Three different parameters were studied for enhanced bacteriocin production using the *L. plantarum* strain, and these required formulating a BBD. This method was preferred for the analysis of a few experimental combinations of the three independent variables to best estimate the optimal bacteriocin yield. In this experimental study, the three key parameters of incubation temperature (A), initial culture medium pH (B), and duration of medium incubation (C) are given in Table 1. Through using the given equations, Design expert version 7 (stat-ease, Minneapolis, MN, USA) provided 17 iterations of the experiments, containing 12 input parameters and 5 simulation studies of the coordinates.

N = 2k (k − 1) + Cp(2)

where

N = total number of experiments, k = number of factors, Cp = number of center points

### 4.5. Statistical Analysis

The bacteriocin yield (Y) in this experiment was obtained by employing correlational research design. The following expression was used to generate the series of specific variables for better bacteriocin production.

Y = β_0_ + β_1_A + β_2_B + β_3_C + β_11_A^2^ + β_22_B^2^ + β_33_C^2^ + β_12_AB + β_13_AC + β_23_BC + ϵ (3)

where

Y = predicted response, β_0_ = constant, A = temperature (°C), B = initial pH, C = incubation time (h), β_1_, β_2_, β_3_ = linear coefficients, β_12_, β_13_, β_23_ = cross product coefficients, β_11_, β_22_, β_33_ = quadratic coefficients, and ε represents random error that is normally distributed with zero mean and constant variance. The positive developments throughout this expression denote the collaborative influence of the parameters, whereas decreases denote an adversarial influence of said factors. The suggested multiple regression analysis [72] effectively estimates the statistical correlation of the responses (Y), in the form of antibiotic production, of all these determinants. Statistically significant correlation with the hypothesis was assessed using ANOVA of the specified research setup. The strengths of fit of the polynomial standard models were determined using the correlation coefficient (R^2^), whereas the relevance of the predictive method and the predictive relevance were determined using an F-test. Two-dimensional contours and three-dimensional area diagrams were used to assess the relationships and major influences of explanatory variables on bacteriocin synthesis. 

The observed data in the experiments are presented as mean ± S.D. SPSS version 25 was used for statistical analysis. One-way analysis of variance (ANOVA) and Duncan’s multiple range tests were used to compare the groups. Differences were considered as significant when *p* < 0.05.

## 5. Conclusions

In the present study, a Box–Behnken design was used to optimize the parameters of the production of antibacterials by *L. plantarum*. The model predicted maximum production of antibacterials at 35 °C, pH 6.5, with an incubation time of 48 h. The statistics revealed that the model, compared to the experimental data, was satisfactorily reliable and precise. The higher coefficient of determination (R^2^ = 0.9983) in ANOVA indicated that the generated quadratic polynomial regression model was adequate. Under optimized conditions, it was possible to obtain a much greater quantity (more than 10-fold) of antibacterials.

Conclusively, RSM with Box–Behnken design turned out to be suitable for optimizing the conditions to obtain antibacterials on a large scale from a unique strain of *L. plantarum.* This could be useful in various health and industrial applications, ranging from food preservatives to discover new alternative-to-antibiotics drugs and new anti-infective biomaterial technologies for orthopedic implants. 

## Figures and Tables

**Table 1:** The coded and actual values of independent variables used in the experiment.

Independent Variables	Coded Values		
	−1	0	+1
A: Temperature (°C)	25	35	45
B: Initial pH	5.5	6.5	7.5
C: Incubation time (h)	24	48	72

**Table 2:** Parameters used for improved bacteriocin production by *Lactobacillus plantarum*.

Runs	A: Temperature (°C)	B: Initial pH	C: Incubation Time (h)	Inhibitory Activity (AU/mL)		Residual Values
				Experimental Value	Predicted Value	
1	25	5.5	48	1250	1115.63	134.37
2	35	6.5	48	650	740.63	−90.63
3	45	7.5	48	450	359.38	90.62
4	35	7.5	72	850	984.38	−134.38
5	45	6.5	24	350	534.38	−184.38
6	35	6.5	48	850	809.38	40.62
7	35	5.5	72	650	690.63	−40.63
8	25	6.5	24	850	665.63	184.37
9	35	6.5	48	900	850	50
10	35	6.5	48	375	281.25	93.75
11	45	5.5	48	450	543.75	−93.75
12	45	6.5	72	550	600	−50
13	25	6.5	72	3550	3530	20
14	35	5.5	24	3400	3530	−130
15	35	6.5	48	3650	3530	120
16	35	7.5	24	3450	3530	−80
17	25	7.5	48	3600	3530	70

**Table 3:** ANOVA result for bacteriocin production by *Lactobacillus plantarum* in the quadratic model.

Variation Source	SS	Df	MS	F Value	*p* Value
Model	2.928 × 10^7^	9	3.254 × 10^6^	120.25	<0.0001
A-temperature	31,250	1	31,250	1.15	0.3182
B-initial pH	1.313 × 10^5^	1	1.313 × 10^5^	4.85	0.0634
C-incubation time	78.13	1	78.13	2.887 × 10^−3^	0.9586
AB	2.500 × 10^5^	1	2.500 × 10^5^	9.24	0.0189
AC	22,500	1	22,500	0.83	0.3921
BC	97,656.25	1	97,656.25	3.61	0.0992
A^2^	7.246 × 10^6^	1	7.246 × 10^6^	267.81	<0.0001
B^2^	8.468 × 10^6^	1	8.468 × 10^6^	312.95	<0.0001
C^2^	1.003 × 10^7^	1	1.003 × 10^7^	370.55	<0.0001
Residual	1.894 × 10^5^	7	27,058.04		
Lack of Fit	1.464 × 10^5^	3	48,802.08	4.54	0.0890
Pure Error	43,000	4	10,750.00		
Corr. total	2.947 × 10^7^	16			
R^2^ = 0.9936	R^2^_Adj_ = 0.9853	R^2^_Pred_ = 0.9182	C.V. % = 10.83	Mean = 1519.12	Std. Dev. = 164.49

## Data Availability

No new data were created or analyzed in this study. Data sharing is not applicable to this article.
